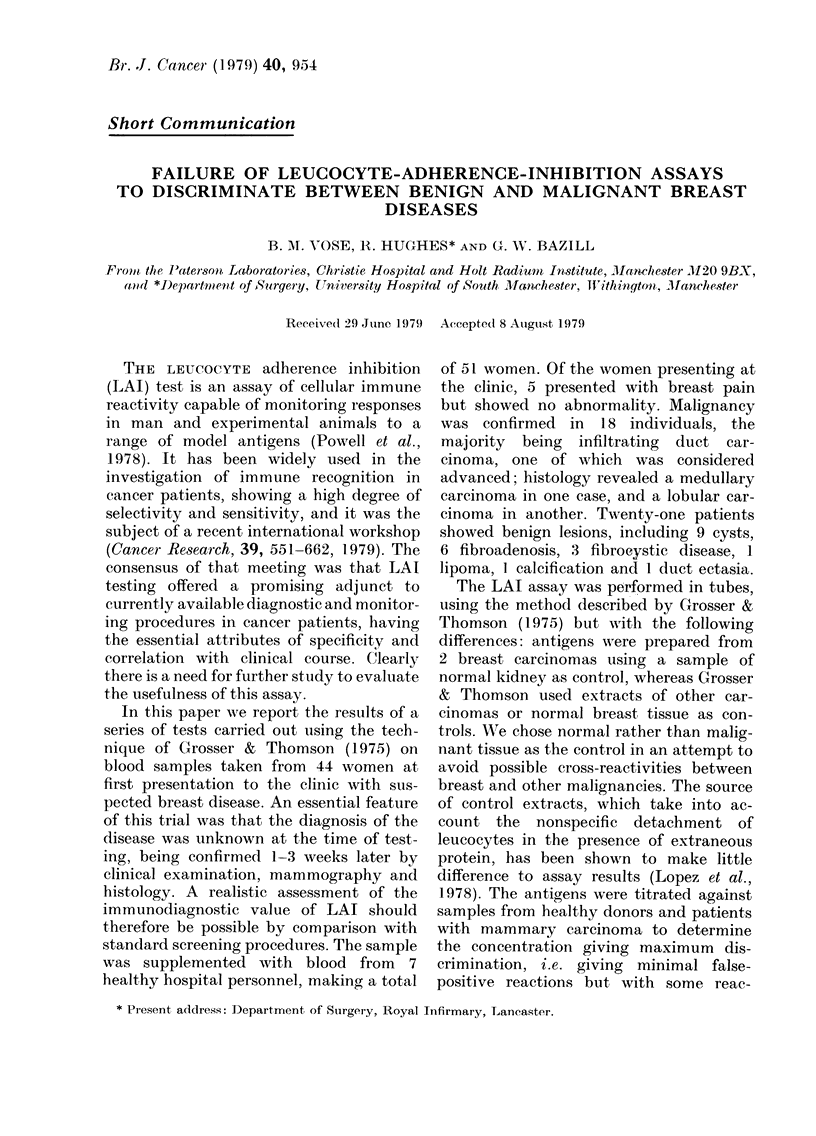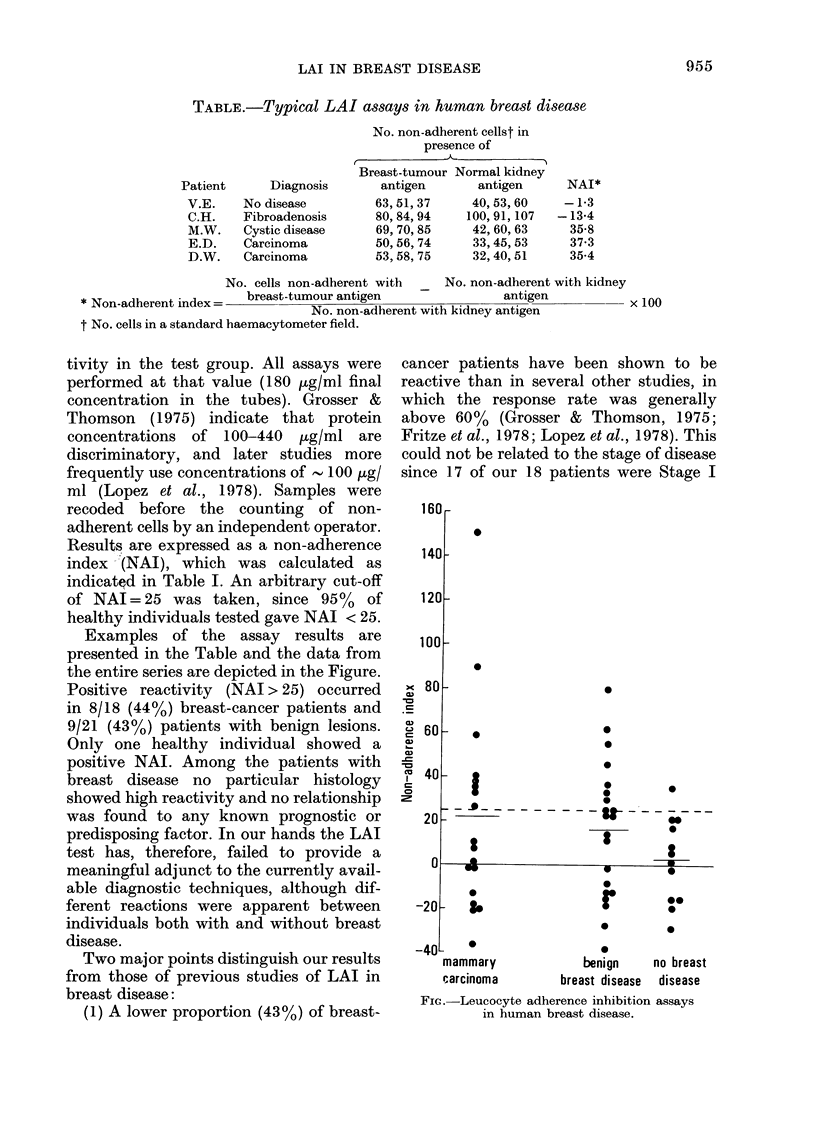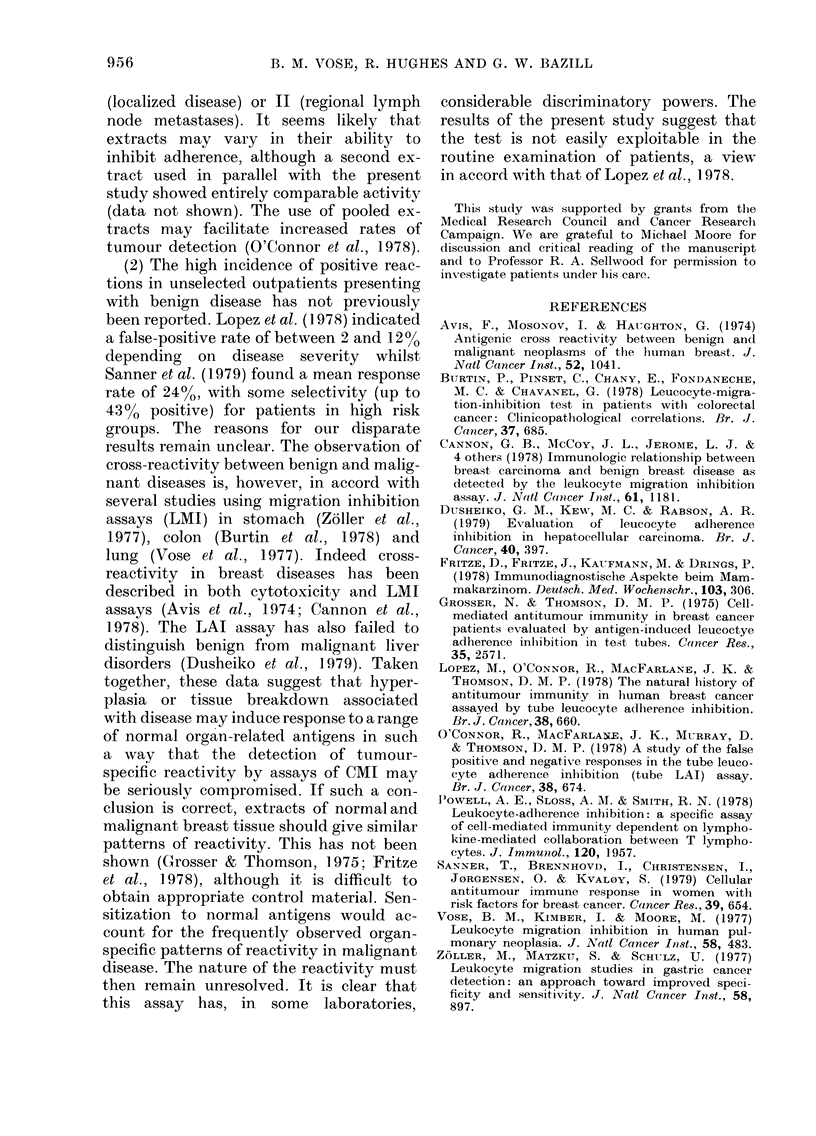# Failure of leucocyte-adherence-inhibition assays to discriminate between benign and malignant breast diseases.

**DOI:** 10.1038/bjc.1979.292

**Published:** 1979-12

**Authors:** B. M. Vose, R. Hughes, G. W. Bazill


Br. J. Cancer (1979) 40, 954

### Short Communication

### FAILURE OF LEUCOCYTE-ADHERENCE-INHIBITION ASSAYS TO DISCRIMINATE BETWEEN BENIGN AND MALIGNANT BREAST DISEASES

B. M. VOSE, R. HUGHES* AND G. W. BAZILL

*From the Paterson Laboratories, Christie Hospital and Holt Radium Institute, Manchester M20 9BX, and *Department of Surgery, University Hospital of South Manchester, Withington, Manchester*

Received 29 June 1979 Accepted 8 August 1979

THE LEUCOCYTE adherence inhibition (LAI) test is an assay of cellular immune reactivity capable of monitoring responses in man and experimental animals to a range of model antigens (Powell *et al.*, 1978). It has been widely used in the investigation of immune recognition in cancer patients, showing a high degree of selectivity and sensitivity, and it was the subject of a recent international workshop (*Cancer Research*, **39,** 551–662, 1979). The consensus of that meeting was that LAI testing offered a promising adjunct to currently available diagnostic and monitoring procedures in cancer patients, having the essential attributes of specificity and correlation with clinical course. Clearly there is a need for further study to evaluate the usefulness of this assay.

In this paper we report the results of a series of tests carried out using the technique of Grosser & Thomson (1975) on blood samples taken from 44 women at first presentation to the clinic with suspected breast disease. An essential feature of this trial was that the diagnosis of the disease was unknown at the time of testing, being confirmed 1–3 weeks later by clinical examination, mammography and histology. A realistic assessment of the immunodiagnostic value of LAI should therefore be possible by comparison with standard screening procedures. The sample was supplemented with blood from 7 healthy hospital personnel, making a total of 51 women. Of the women presenting at the clinic, 5 presented with breast pain but showed no abnormality. Malignancy was confirmed in 18 individuals, the majority being infiltrating duct carcinoma, one of which was considered advanced; histology revealed a medullary carcinoma in one case, and a lobular carcinoma in another. Twenty-one patients showed benign lesions, including 9 cysts, 6 fibroadenosis, 3 fibrocystic disease, 1 lipoma, 1 calcification and 1 duct ectasia.

The LAI assay was performed in tubes, using the method described by Grosser & Thomson (1975) but with the following differences: antigens were prepared from 2 breast carcinomas using a sample of normal kidney as control, whereas Grosser & Thomson used extracts of other carcinomas or normal breast tissue as controls. We chose normal rather than malignant tissue as the control in an attempt to avoid possible cross-reactivities between breast and other malignancies. The source of control extracts, which take into account the nonspecific detachment of leucocytes in the presence of extraneous protein, has been shown to make little difference to assay results (Lopez *et al.*, 1978). The antigens were titrated against samples from healthy donors and patients with mammary carcinoma to determine the concentration giving maximum discrimination, *i.e.* giving minimal false-positive reactions but with some reac-

* Present address: Department of Surgery, Royal Infirmary, Lancaster.

TABLE.—*Typical LAI assays in human breast disease*

| Patient | Diagnosis | No. non-adherent cells† in presence of: Breast-tumour antigen | Normal kidney antigen | NAI* |
|---|---|---|---|---|
| V.E. | No disease | 63, 51, 37 | 40, 53, 60 | −1·3 |
| C.H. | Fibroadenosis | 80, 84, 94 | 100, 91, 107 | −13·4 |
| M.W. | Cystic disease | 69, 70, 85 | 42, 60, 63 | 35·8 |
| E.D. | Carcinoma | 50, 56, 74 | 33, 45, 53 | 37·3 |
| D.W. | Carcinoma | 53, 58, 75 | 32, 40, 51 | 35·4 |

$$\text{* Non-adherent index} = \frac{\text{No. cells non-adherent with breast-tumour antigen} - \text{No. non-adherent with kidney antigen}}{\text{No. non-adherent with kidney antigen}} \times 100$$

† No. cells in a standard haemacytometer field.

tivity in the test group. All assays were performed at that value (180 μg/ml final concentration in the tubes). Grosser & Thomson (1975) indicate that protein concentrations of 100–440 μg/ml are discriminatory, and later studies more frequently use concentrations of ~100 μg/ml (Lopez *et al.*, 1978). Samples were recoded before the counting of non-adherent cells by an independent operator. Results are expressed as a non-adherence index (NAI), which was calculated as indicated in Table I. An arbitrary cut-off of NAI = 25 was taken, since 95% of healthy individuals tested gave NAI < 25.

Examples of the assay results are presented in the Table and the data from the entire series are depicted in the Figure. Positive reactivity (NAI > 25) occurred in 8/18 (44%) breast-cancer patients and 9/21 (43%) patients with benign lesions. Only one healthy individual showed a positive NAI. Among the patients with breast disease no particular histology showed high reactivity and no relationship was found to any known prognostic or predisposing factor. In our hands the LAI test has, therefore, failed to provide a meaningful adjunct to the currently available diagnostic techniques, although different reactions were apparent between individuals both with and without breast disease.

Two major points distinguish our results from those of previous studies of LAI in breast disease:

(1) A lower proportion (43%) of breast-cancer patients have been shown to be reactive than in several other studies, in which the response rate was generally above 60% (Grosser & Thomson, 1975; Fritze *et al.*, 1978; Lopez *et al.*, 1978). This could not be related to the stage of disease since 17 of our 18 patients were Stage I

FIG.—Leucocyte adherence inhibition assays in human breast disease.

(localized disease) or II (regional lymph node metastases). It seems likely that extracts may vary in their ability to inhibit adherence, although a second extract used in parallel with the present study showed entirely comparable activity (data not shown). The use of pooled extracts may facilitate increased rates of tumour detection (O'Connor *et al.*, 1978).

(2) The high incidence of positive reactions in unselected outpatients presenting with benign disease has not previously been reported. Lopez *et al.* (1978) indicated a false-positive rate of between 2 and 12% depending on disease severity whilst Sanner *et al.* (1979) found a mean response rate of 24%, with some selectivity (up to 43% positive) for patients in high risk groups. The reasons for our disparate results remain unclear. The observation of cross-reactivity between benign and malignant diseases is, however, in accord with several studies using migration inhibition assays (LMI) in stomach (Zöller *et al.*, 1977), colon (Burtin *et al.*, 1978) and lung (Vose *et al.*, 1977). Indeed cross-reactivity in breast diseases has been described in both cytotoxicity and LMI assays (Avis *et al.*, 1974; Cannon *et al.*, 1978). The LAI assay has also failed to distinguish benign from malignant liver disorders (Dusheiko *et al.*, 1979). Taken together, these data suggest that hyperplasia or tissue breakdown associated with disease may induce response to a range of normal organ-related antigens in such a way that the detection of tumour-specific reactivity by assays of CMI may be seriously compromised. If such a conclusion is correct, extracts of normal and malignant breast tissue should give similar patterns of reactivity. This has not been shown (Grosser & Thomson, 1975; Fritze *et al.*, 1978), although it is difficult to obtain appropriate control material. Sensitization to normal antigens would account for the frequently observed organ-specific patterns of reactivity in malignant disease. The nature of the reactivity must then remain unresolved. It is clear that this assay has, in some laboratories, considerable discriminatory powers. The results of the present study suggest that the test is not easily exploitable in the routine examination of patients, a view in accord with that of Lopez *et al.*, 1978.

This study was supported by grants from the Medical Research Council and Cancer Research Campaign. We are grateful to Michael Moore for discussion and critical reading of the manuscript and to Professor R. A. Sellwood for permission to investigate patients under his care.

### REFERENCES

AVIS, F., MOSONOV, I. & HAUGHTON, G. (1974) Antigenic cross reactivity between benign and malignant neoplasms of the human breast. *J. Natl Cancer Inst.*, **52**, 1041.

BURTIN, P., PINSET, C., CHANY, E., FONDANECHE, M. C. & CHAVANEL, G. (1978) Leucocyte-migration-inhibition test in patients with colorectal cancer: Clinicopathological correlations. *Br. J. Cancer*, **37**, 685.

CANNON, G. B., MCCOY, J. L., JEROME, L. J. & 4 others (1978) Immunologic relationship between breast carcinoma and benign breast disease as detected by the leukocyte migration inhibition assay. *J. Natl Cancer Inst.*, **61**, 1181.

DUSHEIKO, G. M., KEW, M. C. & RABSON, A. R. (1979) Evaluation of leucocyte adherence inhibition in hepatocellular carcinoma. *Br. J. Cancer*, **40**, 397.

FRITZE, D., FRITZE, J., KAUFMANN, M. & DRINGS, P. (1978) Immunodiagnostische Aspekte beim Mammakarzinom. *Deutsch. Med. Wochenschr.*, **103**, 306.

GROSSER, N. & THOMSON, D. M. P. (1975) Cell-mediated antitumour immunity in breast cancer patients evaluated by antigen-induced leucoctye adherence inhibition in test tubes. *Cancer Res.*, **35**, 2571.

LOPEZ, M., O'CONNOR, R., MACFARLANE, J. K. & THOMSON, D. M. P. (1978) The natural history of antitumour immunity in human breast cancer assayed by tube leucocyte adherence inhibition. *Br. J. Cancer*, **38**, 660.

O'CONNOR, R., MACFARLANE, J. K., MURRAY, D. & THOMSON, D. M. P. (1978) A study of the false positive and negative responses in the tube leucocyte adherence inhibition (tube LAI) assay. *Br. J. Cancer*, **38**, 674.

POWELL, A. E., SLOSS, A. M. & SMITH, R. N. (1978) Leukocyte-adherence inhibition: a specific assay of cell-mediated immunity dependent on lymphokine-mediated collaboration between T lymphocytes. *J. Immunol.*, **120**, 1957.

SANNER, T., BRENNHOVD, I., CHRISTENSEN, I., JØRGENSEN, O. & KVALØY, S. (1979) Cellular antitumour immune response in women with risk factors for breast cancer. *Cancer Res.*, **39**, 654.

VOSE, B. M., KIMBER, I. & MOORE, M. (1977) Leukocyte migration inhibition in human pulmonary neoplasia. *J. Natl Cancer Inst.*, **58**, 483.

ZÖLLER, M., MATZKU, S. & SCHULZ, U. (1977) Leukocyte migration studies in gastric cancer detection: an approach toward improved specificity and sensitivity. *J. Natl Cancer Inst.*, **58**, 897.